# Forensic-psychiatric and medico-legal challenges in euthanasia and assisted suicide for patients with mental disorders: balancing autonomy, vulnerability, and professional responsibility

**DOI:** 10.3389/fpsyt.2026.1835123

**Published:** 2026-06-04

**Authors:** Dalila Tripi, Mario Giantesani, Jan Francesco Arena, Stefano Ferracuti

**Affiliations:** 1 Department of Human Neurosciences, Faculty of Medicine and Dentistry, Sapienza University of Rome, Rome, Italy; 2 Department of Dynamic and Clinical Psychology and Health Studies, Faculty of Medicine and Psychology, Sapienza University of Rome, Rome, Italy

**Keywords:** bioethics, decision making, euthanasia, forensic psychiatry, mental disorders, suicide, assisted, treatment-resistant depression

## Abstract

**Background:**

Euthanasia and assisted suicide (EAS) in individuals with mental disorders represents a complex and controversial area at the intersection of clinical practice, bioethics, and medico-legal evaluation. While suffering is inherently subjective in both psychiatric and somatic conditions, psychiatric contexts are characterized by greater fluctuation, contextual dependence, and prognostic uncertainty, which complicate the assessment of its severity, persistence, and potential reversibility in medico-legal evaluations.

**Methods:**

A structured narrative review of the international literature was conducted, focusing on clinical, ethical, and medico-legal aspects of EAS in psychiatric contexts. Regulatory frameworks and empirical contributions were analyzed to identify key domains and areas of ongoing debate.

**Results:**

Five interconnected domains emerge as central to psychiatric EAS assessment: decision-making capacity, irremediability of suffering, subjective suffering and voluntariness, and safeguard systems. Across jurisdictions, considerable variability exists in legal models and clinical approaches, although a shared emphasis on repeated, multidisciplinary, and well-documented evaluation processes is evident. Persistent challenges include the absence of standardized criteria for irremediability, inter-rater variability in capacity assessment, and the difficulty of distinguishing between psychopathology-related suicidality and well-considered requests for assisted death.

**Discussion:**

The available literature suggests that psychiatric EAS requires particularly careful and structured evaluation processes, given the inherent complexity and variability of mental disorders. Multidisciplinary approaches involving clinical psychiatry, forensic psychiatry, and legal medicine may contribute to improving consistency, transparency, and medico-legal robustness. Longitudinal assessment and detailed reconstruction of clinical history appear to be key elements in supporting reliable decision-making.

**Conclusions:**

Psychiatric EAS remains an evolving and methodologically challenging topic. While uncertainties cannot be fully resolved, structured and multidisciplinary evaluation processes may help enhance the consistency and ethical sustainability of assessments in this context.

## Introduction

Clinical practice in psychiatry is closely associated with suicide prevention and the mitigation of self-harm risk through therapeutic and supportive interventions. The emergence of euthanasia and assisted suicide (EAS) requests from individuals with mental disorders therefore introduces a significant tension within clinical practice, challenging the balance between treatment, respect for autonomy, protection of vulnerability, and professional responsibility. Unlike somatic end-of-life contexts, psychiatric suffering is inherently subjective, often fluctuating, and characterized by prognostic uncertainty, raising complex questions concerning decision-making capacity, irremediability of suffering, and the medico-legal sustainability of assisted death assessments (1–6).

The international landscape reflects markedly different ethical and legal approaches. In the Netherlands and Belgium, EAS for mental disorders has been regulated for over a decade within detailed procedural frameworks requiring multiple independent consultations, documentation of exhaustive treatment attempts, and ex post review by national commissions (3, 7, 8). In Switzerland, assisted suicide is permitted provided it is not motivated by selfish intent. However, this framework is complemented by specific professional guidelines issued by the Swiss Academy of Medical Sciences (SAMS) and the Swiss Medical Association, which are binding for physicians and regulate the clinical assessment process. Furthermore, the Swiss Federal Court has accepted assisted suicide in individuals with mental disorders under strict conditions, emphasizing the need for rigorous evaluation of decision-making capacity and voluntariness. As a result, while non-medical organizations play a role, clinical practice is not solely governed by private protocols but also by established medical and legal standards. In Canada, the planned extension of Medical Assistance in Dying (MAiD) to mental disorders as the sole underlying condition has been repeatedly postponed, reflecting ongoing difficulties in defining clinically reliable and ethically acceptable criteria (9, 10). Conversely, countries such as Italy, France, and Spain adopt precautionary models that exclude mental disorders as sole grounds for access to EAS (11, 12).

Within this heterogeneous context, it could be methodologically essential to distinguish between requests primarily motivated by a mental disorder and those arising in the presence of a severe or terminal somatic condition with psychiatric comorbidity. This distinction has substantial implications for the assessment of decision-making capacity, prognosis, vulnerability, and medico-legal responsibility.

In jurisdictions where psychiatric EAS is permitted, regulatory models rely on reinforced safeguards designed to ensure voluntariness, informed consent, and protection of vulnerable individuals (3, 7). In psychiatric contexts, these safeguards typically include repeated and decision-specific capacity assessments, independent psychiatric consultations, comprehensive reconstruction of treatment history, cooling-off periods, and formal systems of ex post oversight (4, 5, 9). While such measures aim to balance respect for autonomy with the duty of protection and professional responsibility, their practical implementation raises further concerns regarding inter-rater variability, definitional ambiguity, and the limits of prognostic judgment in mental disorders.

The debate can be structured around four interconnected axes. The first concerns decision-making capacity, understood as a task-specific, dynamic construct requiring structured and repeated evaluation (13–15). The second involves the interpretation of “unbearable suffering” and “irremediability,” concepts central to EAS legislation yet particularly fragile in psychiatry, where prognosis is heterogeneous and therapeutic response remains highly individualized (2, 3, 16). In this context, treatment resistance represents a probabilistic rather than definitive condition and cannot be automatically equated with clinical irreversibility; the absence of shared operational definitions risks exposing the notion of irremediability to inconsistent and potentially arbitrary interpretations, with significant ethical and medico-legal implications (17–19).

The third axis concerns the distinction between a stable, deliberative request for assisted death and suicidal ideation associated with psychopathology—a boundary that requires careful, longitudinal, and context-sensitive assessment (1, 20, 21). Finally, the fourth axis addresses medico-legal accountability: in permissive systems, clinicians must ensure rigorous documentation, transparency, and procedural traceability, as errors in evaluation may entail serious professional and legal consequences and ethical implications (3, 22, 23).

Despite an expanding body of international literature, no structured forensic-psychiatric framework currently integrates longitudinal capacity assessment, dynamic interpretation of irremediability, and medico-legal sustainability within a unified operational model (3, 4, 9). The present study aims to address this gap through a structured narrative review of recent evidence and regulatory developments, with the objective of contributing to a more coherent, transparent, and professionally sustainable evaluative framework for psychiatric EAS.

## Materials and methods

We conducted a structured narrative review aimed at critically and comparatively examining the ethical, psychiatric, and medico-legal implications of euthanasia and medically assisted suicide (EAS) in patients with mental disorders. A structured narrative review was considered the most appropriate methodological approach given the ethical, legal, and clinical heterogeneity of the topic, as well as the predominance of conceptual and interdisciplinary contributions that are not fully amenable to systematic quantitative synthesis. Particular attention was devoted to decision-making capacity, the concept of irremediability of psychological suffering, and the safeguard systems adopted in jurisdictions where EAS is regulated or under debate. The methodological approach was informed by the Scale for the Assessment of Narrative Review Articles (SANRA) (24), which was used to enhance transparency, internal consistency, and scientific rigor of the narrative synthesis. In line with its intended purpose, SANRA was not applied as a formal tool for assessing the methodological quality of the included studies, but rather as a framework guiding the structure, clarity, and comprehensiveness of the review process. An extensive literature search was conducted across three primary bibliographic databases: PubMed/MEDLINE, Scopus, and Web of Science. For PubMed, the following query was used: (“euthanasia” OR “physician-assisted suicide” OR “medical assistance in dying” OR “medically assisted suicide”) AND (“psychiatry*” OR “mental disorder*” OR “severe mental illness” OR “treatment-resistant depression”) AND (“ethics” OR “forensic” OR “medico-legal” OR “decision-making capacity” OR “irremediable suffering”). In Scopus, the search was performed using the TITLE-ABS-KEY function, while in Web of Science, the TOPIC (TS=) field was used, applying the same keywords. Articles published between January 2018 and October 2025 were included. This timeframe was selected to capture the most relevant developments following major regulatory changes in countries such as the Netherlands, Belgium, and Canada, as well as ongoing debates in European and North American contexts. Studies were included if they explicitly addressed one or more of the following domains: decision-making capacity, irremediability of psychological suffering, unbearable suffering, suicidal intent, medico-legal aspects, clinical safeguards, or institutional ethical positions. Exclusion criteria comprised studies focusing exclusively on terminal somatic illnesses, non–peer-reviewed or non-scientific contributions, isolated case reports without structured evaluative discussion, purely normative analyzes lacking clinical applicability, studies on non-assisted suicide without methodological relevance to EAS, and articles published in languages other than English or Italian. The initial search yielded 1,463 records: 183 from PubMed, 1,240 from Scopus, and 40 from Web of Science. After removal of 45 duplicates, 1,418 titles and abstracts were screened, leading to the exclusion of 1,350 records primarily due to lack of relevance to psychiatric contexts, exclusive focus on somatic conditions, absence of medico-legal or ethical content, or insufficient scientific rigor. A total of 68 articles were selected for full-text assessment. Following full-text evaluation, 30 articles were excluded due to lack of relevance to decision-making capacity or irremediability, absence of clinical applicability, exclusive focus on dementia without broader psychiatric implications, unavailability of full text, or overall lack of relevance. Selection at the full-text stage was guided by consistency with the predefined domains and by the relevance of each contribution to clinical, ethical, or medico-legal aspects of psychiatric EAS. The final corpus comprised 38 articles (34 from PubMed and 4 from Scopus), while no studies retrieved from Web of Science met the inclusion criteria (see **Tables 1**, **2**). The included studies were analyzed using a thematic extraction matrix designed to allow a structured and comparative interpretation of the evidence. The synthesis was conducted as a comparative narrative analysis, focusing on convergences and divergences across regulatory frameworks, clinical practices, and ethical approaches. No formal quantitative scoring of individual studies was performed, given the heterogeneous and predominantly conceptual nature of the included literature. This approach is consistent with the exploratory and integrative nature of narrative reviews, where the objective is to synthesize interdisciplinary and conceptual evidence rather than to perform quantitative quality appraisal. Study selection and data extraction were performed by two independent reviewers. In cases of disagreement, a third reviewer was consulted, and discrepancies were resolved through collegial discussion.

**Table 1:** Summary of the studies included in the review.

Author	Year	Journal	DOI	Country	Population/focus	Study type	Key points	Role in the review
Kaye	2021	J Am Acad Psychiatry Law	10.29158/JAAPL.210071-21	USA	Ethical-legal debate	Commentary	Critical expansion of PAS criteria	Contextualization of the debate
Kious	2023	Neurol Clin	10.1016/j.ncl.2023.03.002	USA	Neurology and MAiD	Narrative review	Decision-making challenges in dementia	Clinical-ethical framework
Ramos-Pozón et al.	2023	Int J Law Psychiatry	10.1016/j.ijlp.2023.101871	Spain	Spanish EAS legislation	Ethical-legal analysis	Complex requirements for psychiatric patients	Comparative legislation
Pollmächer	2023	Nervenarzt	10.1007/s00115-023-01497-1	Germany	Psychiatry and assisted suicide	Overview	Decision-making capacity and freedom in suicide choice	Role of psychiatry
Rajkumar	2021	Front Sociol	10.3389/fsoc.2021.815233	India	PAS in dementia	Sociocultural analysis	Ethical paradoxes and risks for vulnerable patients	Cultural and vulnerability factors
Conejero et al.	2018	Clin Interv Aging	10.2147/CIA.S130670	France	Older adults, suicide, euthanasia	Review	Risk factors and decision-making in older adults	Advanced age and decisional capacity
Gale & Barak	2020	Australas Psychiatry	10.1177/1039856219878645	NZ/Israel	Psychiatrists’ perspective	Position paper	Critique of euthanasia as a medical act	Professional stance
Nicolini et al.	2020	Psychol Med	10.1017/S0033291720001543	USA/Belgium	EAS for psychiatric disorders	Systematic review of reasons	Eight ethical domains, equality/discrimination	Ethical foundation of debate
Clarke et al.	2021	Int J Law Psychiatry	10.1016/j.ijlp.2021.101747	Ireland	Irish system	Policy review	Risks of the “extended test”	Health policy implications
Colleran & Doherty	2024	Ir J Med Sci	10.1007/s11845-023-03418-2	Ireland	EAS and quality of care	Conceptual analysis	Impact on suicide and vulnerability	Quality of care and systemic risks
Calati et al.	2021	J Psychiatr Res	10.1016/j.jpsychires.2020.12.006	EU	Psychiatric patients requesting EAS	Systematic review	Clinical profile of applicants	Epidemiological evidence
Gristina et al.	2020	Recenti Prog Med	10.1701/3366.33413	Italy	End-of-life care and assisted suicide	Clinical review	Holistic approach and palliative care	Clinical definitions
Willems et al.	2024	Int Rev Psychiatry	10.1080/09540261.2024.2386052	Belgium	Psychiatric services	Health system review	Integration of EAS into psychiatric services	Belgian healthcare context
Cipriani & Di Fiorino	2019	Leg Med (Tokyo)	10.1016/j.legalmed.2019.07.007	Italy	Dementia	Review	Complexity of capacity assessment	Capacity in neurocognitive disorders
Scopetti et al.	2023	Healthcare (Basel)	10.3390/healthcare11101470	Italy	Mental disorders and EAS	Ethical overview	Proportionality, dignity, autonomy	Ethical-legal framework
Nicolini et al.	2023	Psychol Med	10.1017/S0033291722002951	USA/Belgium	TRD and irremediability	Scoping review	Impossibility of predicting irremediability	“Irremediable” construct
Dom et al.	2020	Psychiatr Pol	10.12740/PP/124078	Belgium/Netherlands	Psychiatric E/PAS	Overview	Real-world clinical experience	Evidence from permissive countries
Mukhopadhyay	2021	Asian J Psychiatr	10.1016/j.ajp.2021.102802	India	PAS in dementia	Critical review	Autonomy, depression, impaired judgment	Psychiatric risk variables
Saß & Cording	2022	Nervenarzt	10.1007/s00115-022-01386-z	Germany	Decisional freedom	Forensic commentary	Concept of “freiverantwortlichkeit”	Forensic assessment of capacity
Marijnissen et al.	2022	Front Psychiatry	10.3389/fpsyt.2022.857131	NL/Belgium	Euthanasia in dementia	Narrative review	Due diligence criteria, assessment problems	Applied criteria
Doherty et al.	2022	BJPsych Open	10.1192/bjo.2022.71	Ireland	EAS and non-assisted suicides	Systematic review	No reduction in suicide rates	Epidemiological impact
Jones & Simpson	2024	BJPsych Bull	10.1192/bjb.2024.23	Canada	Prison and forensic populations	Case-based analysis	Ethical-legal conflicts	Vulnerable populations
Bofarull et al.	2025	Am J Geriatr Psychiatry	10.1016/j.jagp.2024.08.002	Spain	Older adults and “life fatigue”	Critical review	Concept of life fatigue	Emerging motivational models
Dalfin et al.	2022	Encephale	10.1016/j.encep.2021.04.013	France	Comparative legislation	Review	Role of psychiatry in assessment	Legislative models
D’cruz	2021	Front Psychiatry	10.3389/fpsyt.2021.700567	India	Identity and autonomy in dementia	Conceptual analysis	Dynamic identity and advance directives	Critique of advance directives
Möller	2021	Int J Psychiatry Clin Pract	10.1080/13651501.2020.1797097	Germany/EU	European EAS	Selected review	Critique of NL criteria expansion	Case escalation concerns
van Mook et al.	2025	Tijdschr Psychiatr	—	Netherlands	Organ donation after EAS	Guideline commentary	Requirement of two psychiatrists	Operational implications
Favron-Godbout & Racine	2023	BMC Med Ethics	10.1186/s12910-023-00971-4	Canada	MAiD-MD	Thematic review	Moral concerns	Layers of moral debate
Sprung et al.	2018	J Palliat Care	10.1177/0825859718777325	Global	PAS/E	Position paper	Five arguments against medicalized EAS	Counter-arguments
Baée et al.	2025	Australas Psychiatry	10.1177/10398562251313917	Australia	E/PAS in non-terminal older adults	Systematic review	70 ethical arguments, majority opposing	Structured ethical arguments
Braun et al.	2022	Philos Ethics Humanit Med	10.1186/s13010-021-00111-z	Germany	Alzheimer’s + assisted suicide	Ethical review	Dignity and value-of-life relationship	Medical ethics
Ramos-Pozón	2025	Monash Bioeth Rev	10.1007/s40592-025-00228-3	Spain	Capacity assessment	Ethical analysis	Narrative approach to capacity	Complex capacity models
Kelly et al.	2019	Palliat Support Care	10.1017/S1478951519000518	Australia	Physicians performing EAS	Mixed qualitative	Emotional impact on clinicians	Debriefing and burnout
Wassiliwizky et al.	2022	Nervenarzt	10.1007/s00115-022-01391-2	Germany	Psychiatrists (DGPPN)	Survey (n=2048)	72% perceive legitimacy in selected cases	Professional attitudes
Komrad et al.	2018	J Clin Psychiatry	10.4088/JCP.18lr12566	USA	Competence assessment	Ethical argument	Competence for EAS deemed non-assessable	Radical critique
Bahji & Delva	2022	J Med Ethics	10.1136/medethics-2020-107133	Canada	MAiD-MD	Review	Possible inclusion of SPMI	Selective inclusion debate
Ghossoub et al.	2018	J Am Acad Psychiatry Law	10.29158/JAAPL.003801-18	USA	Facilitated suicide	Forensic review	Distinction between EAS and facilitation	Legal aspects
Gouveia	2025	Death Stud	10.1080/07481187.2024.2361761	Portugal	MAiD after new law	Review	Decision-making capacity as decision-specific	Proposed capacity model

**Table 2:** SANRA evaluation of included studies.

Author (year)	Study type	Total SANRA score	Quality rating	Methodological notes
Schweren (25)	Retrospective study	11	High	Excellent documentation and methodological rigor
Pronk (26)	Qualitative study	10	High	Rigorous triangulation methodology
Evenblij (27)	Cross-sectional study	9	Moderate	Valid design with partial representativeness
Calati (3)	Systematic review	12	High	Excellent methodological quality
Nicolini (2)	Bioethical review	10	High	Strong theoretical framework
Grassi (28)	Narrative review	9	Moderate–High	Clear and structured presentation
Hachtel (29)	Forensic qualitative study	10	High	Strong psychiatric–forensic relevance
Kupsch (30)	Systematic review	11	High	Validation of decision-making capacity assessment tools
Peereboom (31)	Normative analysis	8	Moderate	Robust within legal context
Italian Constitutional Court Ruling No. 242/2019	Judicial ruling	n/a	—	Included for normative reference value

*(Scores range from 0 to 2; maximum score = 12)*.

This review focused primarily on peer-reviewed journal articles indexed in major bibliographic databases. Consequently, relevant contributions published in books and book chapters were not systematically included. While such sources may provide valuable theoretical and contextual insights, particularly in the field of bioethics, the decision was made to prioritize indexed scientific literature to enhance methodological consistency and reproducibility. This may have resulted in the exclusion of some relevant conceptual contributions.

### Considerations on risk of bias

Given the predominance of conceptual, ethical, and normative contributions in this field, substantial heterogeneity in study design and an inherent degree of interpretative bias were anticipated. This variability reflects the interdisciplinary nature of the topic, where clinical, ethical, and legal perspectives converge without uniformly standardized methodologies. To mitigate subjectivity, a structured thematic extraction framework was adopted, enabling systematic comparison across studies and enhancing the consistency and transparency of the narrative synthesis. In addition, study selection and data extraction were performed independently by two reviewers, with discrepancies resolved through collegial discussion, further reducing the risk of individual interpretative bias. While a formal quantitative risk-of-bias assessment was not applicable due to the predominantly conceptual nature of the included literature, efforts were made to ensure balanced representation of perspectives and methodological transparency throughout the review process.

## Results

The analysis of the 38 selected articles provides a heterogeneous yet partially convergent picture of EAS in individuals with mental disorders. To enhance clarity and comparability, key findings are summarized in **Table 3**, outlining regulatory frameworks across jurisdictions and core clinical and medico-legal dimensions.

**Table 3:** Core clinical and medico-legal dimensions in psychiatric EAS assessment.

Domain	Description	Key challenges	Implications
Decision-making capacity	Ability to understand, appreciate, reason, and express a choice	Fluctuation, influence of psychopathology	Requires structured and repeated assessment
Irremediability	Absence of reasonable therapeutic alternatives	Prognostic uncertainty, evolving treatments	Dynamic and case-specific evaluation
Subjective suffering	Patient-reported unbearable psychological suffering	Lack of objective measures	High inter-rater variability
Voluntariness	Free, informed, and stable decision	External and internal pressures	Requires longitudinal verification
Longitudinal assessment	Stability of the request over time	Changes in motivation and clinical condition	Essential procedural safeguard

The following tables synthesize the main findings to facilitate cross-jurisdictional and conceptual comparison.

Overall, the methodological quality of the analyzed studies was judged to be moderate to high, based on qualitative appraisal of study design, clarity of argumentation, and consistency of reported findings. Empirical investigations—particularly those originating from the Netherlands—as well as systematic reviews demonstrated greater methodological rigor and argumentative clarity. In contrast, purely normative or theoretical contributions, not supported by empirical data, displayed more heterogeneous analytical depth. This variability reflects the interdisciplinary and evolving nature of the field, in which clinical, ethical, and legal dimensions intersect in complex and sometimes unresolved ways. The synthesis identified four pivotal and closely interconnected domains: (i) decision-making capacity; (ii) irremediability of the disorder; (iii) subjective suffering and voluntariness of the request; and (iv) safeguard systems and legal models applied in psychiatric contexts. Together, these dimensions form the core evaluative architecture underpinning the body of examined studies (2–4).

Literature from the Netherlands and Belgium, the two countries with the longest-standing regulation of EAS for mental disorders, describes a system characterized by reinforced safeguards and multilayered oversight. Requests are evaluated through a structured process that includes at least one, and often two, independent consultations (with mandatory psychiatric involvement), detailed documentation demonstrating that all “reasonably available” therapeutic options have been explored, and ex post review by national committees (the Regional Review Committees in the Netherlands and the Federal Commission in Belgium) to verify compliance with statutory criteria. Despite procedural differences in implementation, this architecture represents the most frequently cited and comparatively stable reference model in the international literature (3, 7, 8).

Switzerland follows a distinct approach centered on assisted suicide rather than euthanasia. Article 115 of the Swiss Penal Code prohibits assistance motivated by selfish intent, while permitting it within associations that operate according to internally developed protocols. In this setting, the assessment of decision-making capacity assumes central importance and is typically entrusted to independent psychiatrists tasked with verifying voluntariness, absence of coercion, and adequate understanding of the decision (9).

The Canadian framework is currently in transition. The planned extension of Medical Assistance in Dying (MAiD) to individuals with mental disorders as the sole underlying condition—initially scheduled for 2024 and postponed to 2027—has generated sustained academic and institutional debate. Concerns focus on the need for extended observation periods, stronger evidentiary standards in non-terminal cases, and the adequacy of safeguards. Recent Canadian contributions emphasize prolonged assessment models, multidisciplinary oversight, and repeated verification of decisional stability (9, 10).

In Italy, Constitutional Court ruling no. 242/2019 reflects a markedly restrictive position. Assisted suicide is permitted only in narrowly defined circumstances involving irreversible physical illness and dependence on life-sustaining treatments, thereby excluding primarily psychological suffering. Consequently, the debate on psychiatric EAS remains predominantly theoretical, bioethical, and medico-legal (4, 9). Across these diverse regulatory landscapes, a clinically and medico-legally significant distinction consistently emerges between requests motivated solely by a mental disorder and those arising in the context of severe or irreversible somatic conditions. Although rarely codified explicitly in legal texts, this differentiation substantially influences assessments of decision-making capacity, prognostic evaluation, and the interpretation of irremediability (4, 5, 9). Despite notable regulatory differences, the international literature converges on a shared triad of evaluative criteria (decision-making capacity, irremediability, and voluntariness) that constitutes the ethical and legal core of any admissible procedure. These core clinical and medico-legal dimensions are summarized in **Table 4**. Across all examined systems, there is agreement on the need to document the patient’s treatment history in a traceable manner, to conduct repeated assessments over time, and to establish multidisciplinary evaluation processes (2, 3, 7).

**Table 4:** Comparative overview of regulatory frameworks for psychiatric EAS.

Country	Permitted for mental disorders	Core eligibility criteria	Safeguards	Notes
Netherlands	Yes	Decision-making capacity, unbearable suffering, no reasonable alternatives	Multiple independent psychiatric evaluations, due diligence criteria, review committees	Most established model
Belgium	Yes	Decision-making capacity, suffering, treatment exhaustion	Independent assessments, federal review commission	Similar to Netherlands
Switzerland	Yes (assisted suicide only)	Decision-making capacity, absence of selfish motive	Psychiatric evaluation often required	Non-medical involvement possible
Canada	Not yet (postponed to 2027)	Eligibility criteria under debate	Proposed reinforced safeguards, multidisciplinary assessment	Ongoing controversy
Italy	No	Psychiatric conditions excluded as sole criterion	Constitutional Court limitations	Restrictive model
Spain	No (psychiatric conditions alone)	Serious somatic illness required	Formal legal procedure	Precautionary approach
France	No	Not permitted	—	Highly restrictive

However, the theoretical framework often clashes with real-world challenges. The primary controversy lies in defining irremediability, a concept that remains elusive in mental health care, where treatment responses vary widely between individuals and the biological basis of disorders does not allow for clear cause–effect relationships to support objective evaluation of treatment effectiveness. In this context, the emergence of new therapeutic options, such as transcranial neuromodulation, ketamine/esketamine treatments, psychedelic-assisted psychotherapies, specific psychotherapies, and, in some areas, new therapeutic frontiers have further complicated the possibility of deeming a mental disorder definitively untreatable. Several authors argue that irremediability cannot be assessed solely on a historical basis but must also consider the potential availability of innovative interventions (3, 16).

Nonetheless, the literature points out that the theoretical availability of such options does not necessarily imply clinical accessibility or patient responsiveness, rendering the judgment of irremediability inevitably complex, dynamic, and highly case-specific (2, 3, 16).

Another recurring theme concerns the assessment of psychological suffering. Unlike terminal physical suffering, mental suffering often lacks universally accepted objective indicators and must be evaluated through a combination of clinical judgment, patient reported experience, longitudinal observation, and interdisciplinary discussion. Nevertheless, suffering is always an intrinsically subjective experience, which is always present when it comes to EAS requests. This contributes to substantial inter-rater variability, particularly in affective and personality disorders characterized by fluctuating symptomatology (2, 3).

Empirical data, particularly from Dutch cohorts, reveal relevant patterns: requests from younger individuals rarely lead to approval, and a high proportion of applications are withdrawn or rejected during the evaluation process, often due to changes in clinical condition or personal motivation. Reports of suicidal behavior during the assessment process further underscore the need for risk mitigation strategies, close monitoring, and robust therapeutic alternatives before considering EAS (3, 21).

The clinical processes described in the literature outline a structured ethical-legal pathway in jurisdictions where psychiatric EAS is permitted. This pathway typically begins with a voluntary and informed request, followed by verification of the absence of coercion. Decision-making capacity is assessed through repeated clinical evaluations, often using the four-abilities model and tools such as the MacArthur Competence Assessment Tool for Treatment (MacCAT-T), although alternative instruments are used in specific national contexts. For instance, in Switzerland, the U-Doc instrument is widely adopted in clinical practice to assess decision-making capacity, alongside collateral information from family or caregivers (13–15, 24). Subsequent steps include detailed reconstruction of treatment history, evaluation of irremediability and suffering, independent consultations, a cooling-off period, and final review procedures by competent authorities (3, 7, 8). While implementation varies across countries, this model represents the most advanced attempt to reconcile autonomy, protection, and professional responsibility in psychiatric EAS (2, 3, 9).

Overall, the results of the review portray a landscape in which EAS for mental disorders is characterized by high clinical sensitivity, ethical complexity, and regulatory variability (3, 4, 9). Although the literature aligns on key principles (the centrality of decision-making capacity, the need to verify disorder irremediability, and the requirement for structured, documented, and supervised evaluative processes) significant differences remain in the applied criteria, assessment methods, and clinical-legal outcomes (2, 3, 7). This heterogeneity reflects not only divergences in legal systems but also varying conceptions of psychological suffering, vulnerability, and the role of psychiatry at the end of life (4, 9).

Within this context, the literature shows that many critical issues remain unresolved: the operational definition of irremediability in psychiatry, the limited availability of validated and context-specific standardized tools for assessing decision-making capacity in EAS contexts, and the distinction between suicidality associated with active psychopathology and autonomous, well-considered requests for assisted death (1–3, 16). The available evidence indicates that decision reliability tends to increase when assessments are conducted longitudinally, involve multidisciplinary input, and are supported by detailed clinical documentation (3, 7, 21). Across the analyzed literature, recurring concerns emerge regarding the absence of shared forensic frameworks, variability in clinical criteria, limited validation of specific assessment tools in EAS contexts, and inconsistencies in regulatory infrastructures (4, 9). Several contributions highlight that only a minority of jurisdictions currently provide sufficiently articulated legal references and structured evaluative systems to support coherent psychiatric EAS assessment (9, 10). The need for clearer conceptual definitions, procedural transparency, and interdisciplinary dialogue is repeatedly emphasized within the literature as a condition for enhancing consistency, accountability, and ethical sustainability in systems where psychiatric EAS is permitted (2, 3, 9).

## Discussion

EAS in patients with mental disorders represents one of the most controversial domains in contemporary bioethics, situated at the intersection of the foundational principles of medicine: autonomy, beneficence, non-maleficence, and justice. In mental health contexts, these principles interact dynamically rather than hierarchically, making each evaluative process intrinsically complex and resistant to rigid standardization (2, 3, 9). The principle of justice acquires particular relevance, as access to EAS must be examined in light of potential socioeconomic disparities, unequal access to treatment, and structural care deficits that may influence the formulation of the request itself (4, 5, 9).

Psychiatry’s historical commitment to suicide prevention and life preservation through longitudinal and adaptive therapeutic interventions further intensifies the need for a clear ethical and legal framework (1, 20, 21). The emergence of assisted death requests from patients with mental disorders therefore challenges the epistemic and professional foundations of psychiatric practice, confronting clinicians with a potential conflict between the duty to protect life and the legitimacy of a sustained request for death (1, 2, 6). In this context, it is important to recognize that physicians have a duty not only to protect life but also to alleviate suffering. Such tension requires a structured reflection that integrates clinical, ethical, legal, and medico-legal dimensions rather than isolating them.

A central issue in the debate is decision-making capacity. In the context of psychiatric EAS, the principle of autonomy must be interpreted with particular care and methodological rigor (13, 14). Autonomy in psychiatry is far from binary from a clinical and neurobiological perspective; however, from a legal standpoint, decision-making capacity is typically conceptualized as dichotomous (present or absent), creating a potential tension between clinical complexity legal categorization. It is a complex, dynamic, and layered construct shaped by the patient’s clinical condition at the time of evaluation, relational environment, and individual history (2, 3, 13). Numerous authors, including Pollmächer, Saß and Cording, Ramos-Pozón, and Komrad, agree that decision-making capacity cannot be automatically inferred from the presence of a psychiatric diagnosis; rather, it must be assessed methodically, specifically for the decision at hand (decision-specific), contextualized in time (time-specific), and grounded in observable and documentable clinical elements (6, 11, 13–15, 32, 33). In this regard, the four-abilities model proposed by Appelbaum and Grisso, understanding, appreciation, reasoning, and expression of a choice, serves as a fundamental conceptual framework for assessing decision-making capacity, although it exhibits notable limitations when applied to end-of-life decisions (13, 15). The most commonly used tool, the MacCAT-T, was in fact developed to assess consent to treatment, not irreversible decisions involving the intentional acceptance of one’s own death (15). For this reason, a growing body of literature advocates for supplementing the classical criteria with additional dimensions such as the value-consistency of the choice, motivational stability over time, and resilience of the decision to psychopathological and contextual fluctuations (2, 3, 6). This perspective reinforces the idea that evaluating decision-making capacity in psychiatric EAS cannot be reduced to a one-time certification, but must be conceived as a structured, repeated clinical-evaluative process embedded in a multidisciplinary framework that balances the protection of autonomy with the safeguarding of vulnerability (3, 7, 13–15).

Closely tied to decision-making capacity is the concept of irremediability of psychological suffering, arguably the most theoretically complex issue in the entire debate. As noted by Appelbaum, even in somatic medicine the assessment of suffering and irremediability involves significant degrees of uncertainty and value judgment, highlighting that these challenges are not unique to psychiatric conditions but inherent to end-of-life decision-making more broadly (34). However, in psychiatric disorders these difficulties are further amplified by non-linear clinical trajectories, high inter-individual variability in treatment response, and the absence of reliable prognostic markers (3, 16). The constant evolution of therapeutic options makes it particularly difficult to assign a definitive and irreversible character to psychological suffering, necessitating a prudent, probabilistic, and necessarily case-specific judgment with important ethical and medico-legal implications (5, 16). An additional layer of complexity involves considering eventual differences between suicidal ideation or suicidal behavior associated with active psychopathology and a well-considered request for EAS. In psychiatry, in the categorical system of the DSM, suicidal ideation can be considered a symptom (e.g. major depressive disorder), therefore to which it may represent the direct expression of a mental disorder. On the other hand, the literature on suicide attributes to suicidal ideation the value of the ultimate expression of unbearable mental pain, therefore not pathological in the strict sense and present in many contexts of mental and physical suffering. In this sense, the evaluation of EAS requests, although adopting both perspectives the result can be the search to voluntary death, must consider possible differences in terms of intentionality, evaluative context, and professional responsibility (1, 2, 21). This could help to identify useful references to guide the examination of EAS requests. Conflating these distinctions risks both medicalizing suicide and pathologizing every EAS request, thus obstructing a fair, rigorous, and clinically grounded assessment (1, 6). Recent contributions, including Marckmann and Pollmächer (35), have further emphasized the ethical tension between respect for autonomy and the need to protect vulnerable individuals, particularly in relation to the assessment of irremediability and the distinction between psychopathology-driven suicidality and well-considered requests for assisted death.

Within this framework, psychiatry should assume a role not of *a priori* exclusion but of clinical and ethical-legal safeguarding. The psychiatrist contributes to evaluating decision-making capacity, motivational coherence, will stability, and clinical history, operating within a structured process that protects both patients and professionals (3, 7, 22). This safeguarding role is particularly critical in systems where EAS is permitted, as evaluative errors may lead to irreversible consequences and entail serious legal and ethical repercussions (22, 23). In this regard, the proposal of a dedicated assessment commission is not merely a procedural formality, but a substantive safeguard aimed at reducing decisional variability, distributing professional responsibility, and ensuring a balanced consideration between respect for individual autonomy and protection of psychological vulnerability (3–5, 9). Based on the findings of this narrative review, a possible operational structure may include at least two psychiatrists, one of whom should be independent, responsible for assessing decision-making capacity, motivational coherence, and longitudinal clinical history. This process should be complemented by the involvement of a forensic physician, tasked with ensuring procedural correctness, traceability, and medico-legal sustainability, as well as, where appropriate, a specialist in relevant somatic conditions to evaluate prognosis, quality of life, and remaining therapeutic options (36–39). Within this multidisciplinary framework, forensic psychiatry operates in close integration with forensic medicine, while maintaining a distinct role focused on the structured evaluation of mental states in medico-legal settings. It differs from clinical psychiatry in that its primary role is not therapeutic, but evaluative, providing structured, evidence-based assessments with particular emphasis on decision-making capacity, responsibility, and the medico-legal sustainability of clinical judgments. In this configuration, forensic medicine assumes a central function: not merely certifying procedural steps, but ensuring coherence between clinical evaluations, applicable legal criteria, and supporting documentation, thereby safeguarding the overall medico-legal sustainability of the evaluative process (4, 22, 23). Each member of the commission should produce an independent, reasoned report, followed by a formally recorded collegial decision (3, 7, 8). However, the establishment of such commissions also raises important considerations. On the one hand, there is a potential risk of diffusion of responsibility, whereby individual accountability may become diluted within collective decision-making processes. On the other hand, it is essential to consider the need for formal mechanisms of appeal or review in cases where the individual disagrees with the outcome of the assessment. The absence of such safeguards could lead to concerns regarding arbitrariness and procedural fairness. Such a structure enhances transparency, distributes professional responsibility, and reduces decisional variability, while also mitigating the psychological burden placed on individual clinicians in high-stakes end-of-life contexts (22, 40). Particular caution is warranted in relation to requests from young adults. As noted by Schweren and other Dutch authors, approvals in this age group are rare and typically subject to heightened evaluative thresholds, given the fluctuating nature of psychological suffering in youth and its responsiveness to relational and social interventions (3, 7). The greater neuropsychiatric plasticity and higher likelihood of clinical improvement in younger individuals support the application of stricter evaluative criteria and intensive therapeutic efforts prior to any consideration of EAS (17–20, 41). From a comparative perspective, the literature delineates at least two predominant bioethical orientations: a liberal–autonomist model, exemplified by the Netherlands and Belgium, which prioritizes self-determination, and a precautionary model, reflected in jurisdictions such as Italy, which emphasizes vulnerability protection and minimization of evaluative error (4, 7–9). Within the autonomist perspective, some legal frameworks—most notably the 2020 ruling of the German Federal Constitutional Court—have advanced an autonomy-based approach, according to which the right to a self-determined death does not depend on the presence of illness, suffering, or treatment resistance, but rather on the individual’s capacity to make a free, informed, and stable decision. This perspective differs significantly from models requiring additional clinical criteria such as irremediability and unbearable suffering. While it reinforces the primacy of individual autonomy, it also raises complex clinical and medico-legal concerns, particularly in psychiatric contexts, where decisional capacity may be influenced by fluctuating psychopathology and vulnerability. Rather than representing mutually exclusive paradigms, these models may converge through the implementation of robust safeguards and multidisciplinary assessment processes capable of balancing autonomy and protection (3, 5, 9). Additional ethical, clinical, and medico-legal perspectives emerging from recent literature further support the need for structured and multidisciplinary approaches in psychiatric EAS assessment (31, 42–52). While decision-making capacity remains the primary evaluative criterion, the clinical context in which the request arises may significantly influence the degree of uncertainty, the complexity of the evaluation, and the associated medico-legal implications. For this reason, it remains analytically useful to distinguish between requests primarily motivated by mental disorders and those arising in the context of severe somatic illness with psychiatric comorbidity, that in certain framework might be considered a reason for exclusion *a priori*.

### Euthanasia and medically assisted suicide in mental disorders as the sole motivation for the request

When the request for EAS is motivated solely by a mental disorder, without the presence of a serious or irreversible somatic condition, the clinical, ethical, and medico-legal challenges become significantly amplified (3–5, 9). In such cases, the international literature suggests that a higher and specific evaluative threshold is required, not as a form of discrimination, but as a proportional response to increased diagnostic and prognostic uncertainty, variability in treatment response, and the need for careful evaluation of suicidality and chronic suicidality possibly emerging in the context of active psychopathology (1–3, 21). Some authors emphasize the importance of distinguishing between clinical psychological suffering, existential suffering, and suffering secondary to social or relational factors (37, 53, 54). While the former clearly falls within the scope of psychiatric assessment, the latter two call for supportive, palliative, and social interventions and cannot be automatically considered indicators of clinical irremediability (4, 5, 53). A key issue concerns the assessment of decision-making capacity in severe mental disorders, particularly in schizophrenia spectrum disorders, mental disorders with psychotic features and severe mood disorders. Available evidence indicates that these conditions do not automatically entail incapacity but may selectively impair components of decision-making, particularly appreciation and reasoning (13–15). In severe depressive disorders, for instance, negative cognitive biases, hopelessness, and rigidly pessimistic future outlooks may distort the evaluation of therapeutic alternatives and the perception of reversibility of suffering (14, 17–19). As a result, the automatic exclusion of patients with severe mental disorders would constitute an unjustified presumption of incapacity, incompatible with the forensic principle of decision-specific and time-specific capacity assessment (13–15, 33). At the same time, the literature suggests that in such cases, the assessment must be more comprehensive, repeated, and supported by structured tools and in-depth clinical analysis of the psychopathological history (3, 6, 32). The distinction between suicidal ideation or suicidal behavior associated with active psychopathology and a well-considered EAS request is one of the most delicate issues in this context. Numerous studies highlight that a significant proportion of patients requesting EAS for mental disorders have a history of suicidal ideation or attempts, raising the concern that the desire to die may reflect pathology rather than an autonomous, deliberative choice (1, 20, 21). This reopens a fundamental question about the role of psychiatry: how to reconcile the inherent mission of suicide prevention with the assessment of the legitimacy of an assisted death request? The literature suggests that the answer lies not in *a priori* exclusion but in a structured, longitudinal, and shared evaluative process capable of distinguishing suicidal ideation associated with active psychopathology from a thoughtful and stable decision over time (1–3). Without this distinction, there is a high risk of conflating two clinically and ethically distinct phenomena (6). Suicidal ideation in psychiatric contexts often emerges in association with acute psychological suffering or active psychopathology; however, its clinical meaning must be assessed carefully and should not be reduced to a single, uniform category (20, 21). Ethical systems must be prepared to acknowledge both suffering and intentionality as meaningful, requiring careful evaluation and a willingness to recognize the full dignity of each case (2, 4, 9). This further underscores the inadequacy of standardized criteria alone, without critical, collegial, and empathetic reflection guiding EAS evaluations for mental disorders (3–5). The concept of irremediability in mental suffering is particularly problematic in this context. Unlike terminal somatic conditions, irremediability in mental disorders cannot be defined in absolute terms; it is inherently probabilistic, and its assessment relies on either subjective experience or objective indicators of suffering (e.g., individual functioning) (3, 16). Moreover, the continuous evolution of therapeutic options could make some of the different definitions of treatment-resistance obsolete (17–19). For instance, the concept of treatment resistance, often used as an implicit criterion for irremediability, is currently insufficient if not updated to reflect modern multimodal strategies, including neuromodulation, intensive psychotherapies, and integrated psychosocial interventions (16–19). Failure to respond to a limited sequence of pharmacological treatments cannot be equated with clinical irreversibility without risking premature and unfounded decisions (3, 16). At present, a growing body of evidence supports the need for more integrated clinical frameworks, which would also benefit forensic psychiatry and legal medicine (17–19). In light of these factors, documented longitudinal assessment emerges as a key safeguard in psychiatric contexts (3, 7, 8). The literature stresses the importance of reconstructing the patient’s entire clinical pathway in detail, including treatment history, adherence, responses, side effects, interruptions, and reasons for ineffectiveness (3, 16). This reconstruction is not only clinically important but also crucial from a medico-legal perspective, demonstrating that the evaluation of irremediability and treatment proportionality was conducted rigorously and non-arbitrarily (4, 22, 23). Longitudinal assessment also allows clinicians to track the stability or evolution of the patient’s will over time, reducing the risk of making irreversible decisions during acute psychopathological episodes or transient crises (3, 7). It is therefore recommended that EAS requests motivated solely by mental disorders be assessed within dedicated multidisciplinary commissions serving as key clinical and ethical-legal safeguards (3–5, 9). In this specific context, the commission’s function is not merely to verify formal eligibility criteria but to conduct a reinforced prudential evaluation aimed at mitigating the risk of irreversible errors arising from prognostic uncertainty or psychopathological interference (1, 3, 6). An adequate assessment structure should include at least two independent psychiatric evaluations, with particular attention to the longitudinal clinical history and the coherence and persistence of the request over time; the involvement of a forensic physician to ensure procedural integrity, traceability, and legal-medical viability; and, when appropriate, input from other specialists able to assess remaining therapeutic options or relevant clinical factors (4, 22, 23). In this configuration, the commission serves as an institutional forum for integrating expertise, more effectively balancing autonomy and protection than decisions made by individual clinicians (3, 7, 9).

Family involvement represents another sensitive aspect of this scenario. While relatives may provide valuable information on clinical history, symptom progression, and the consistency of the request with the patient’s life values, their involvement poses non-negligible medico-legal risks (40, 54). Relational conflict, guilt, implicit expectations, or unintentional pressure may influence the decision-making process (40). Therefore, the literature advises that family input be considered informative and collateral rather than decisive, requiring clear delineation of roles and explicit verification of absence of coercion, preferably within a structured collegial context (3–5).

Taken together, these elements underscore that EAS in mental disorders as the sole motivation for the request demands the utmost caution, requiring a higher evaluative threshold than other clinical contexts (3, 4, 9). The combination of prognostic uncertainty, the risk of overlap with pathological suicidality, the fragile nature of irremediability, and the complexity of capacity assessment calls for strengthened safeguards, documented longitudinal evaluations, and multidisciplinary decision-making processes (2, 3, 7, 16). In the absence of these conditions, the risk of irreversible errors and ethically or medico-legally problematic outcomes becomes unacceptably high (4, 22, 23).

### Euthanasia and medically assisted suicide in the presence of somatic comorbidity and non-causative psychiatric disorder

A distinct clinical and ethical scenario arises when EAS requests are made by patients with serious, irreversible, or terminal somatic illnesses who also present with a current or historical psychiatric comorbidity that is not the primary cause of the request (37–39, 54). In such cases, the literature emphasizes the need to avoid automatic or discriminatory approaches based solely on psychiatric diagnosis, advocating for individualized, decision-specific capacity assessments (2, 4, 9). One widely shared principle emerges clearly: a psychiatric history should not constitute grounds for excluding access to EAS when the request is motivated by a serious somatic condition and the individual retains intact decision-making capacity (4, 9). Capacity is not diagnosis-specific, but rather decision-specific and time-specific; it must be assessed in relation to the specific decision at hand and the patient’s current clinical condition (13–15, 33, 55, 56). An individual with a past diagnosis of major depressive disorder, bipolar disorder, or another mental illness, now in full remission or clinically stable, may demonstrate decision-making capacity fully comparable to someone with no psychiatric history (13–15, 55, 56). *A priori* denial of access to EAS in such cases would amount to a presumption of permanent incapacity, lacking clinical justification and contravening principles of equity, justice, and non-discrimination (2, 4, 9).

The literature highlights how automatic exclusion of psychiatric patients, even when their diagnosis is not causally related to the end-of-life request, risks institutionalizing stigma (1, 4, 6). This approach, unsupported by scientific evidence, relies on a dichotomous conception of the mind-body relationship that does not reflect clinical reality, where somatic and psychological suffering often intertwine (37, 54). In medico-legal contexts, this risks unjustifiably constraining autonomy in contradiction to the obligation for individualized evaluation in high-impact decisions (4, 22, 23).

In this scenario, psychiatry plays a primarily safeguarding role. The psychiatrist is not tasked with “validating” the EAS request based on diagnosis, nor with exercising a blanket veto, but with rigorously assessing current decision-making capacity, emotional stability, absence of active psychopathology that might interfere with decision-making, and consistency of the request with the patient’s life values (13–15, 32, 33). This assessment must be embedded in a structured and documented process that considers the longitudinal psychiatric history and the disorder’s course over time (3, 7). Here, the function of a multidisciplinary commission differs from that in cases where mental disorder is the sole motivation: its role is not to raise the evaluative threshold, but to prevent arbitrary exclusions or discriminatory practices based on psychiatric diagnosis (3–5). The commission thereby ensures fairness in the decision-making process, affirming that psychiatric comorbidity is evaluated according to its actual clinical relevance and not used as an automatic barrier (9). The presence of a serious somatic condition introduces additional evaluative elements, such as impact on quality of life, care burden, proportionality of remaining therapeutic options, and overall prognosis (36–39). In these cases, the participation of specialists in the somatic illness and clinicians such as internists or geriatricians allows for a more comprehensive assessment of the clinical context, reducing the risk of overestimating or unjustifiably disqualifying mental suffering (36, 38, 39). Multidisciplinary evaluation should help ensure a proper evaluation of decision-making capacity (13–15).

Within the commission, the psychiatrist’s input complements that of other specialists and the forensic physician, who verifies procedural coherence, clinical documentation, and the medico-legal viability of conclusions reached (4, 22, 23). In this structure, the commission does not replace individual clinical evaluation but strengthens it, enhancing transparency, traceability, and ethical-legal defensibility (3, 7, 9).

A key concern, noted by several contributions, is the opposite risk to that seen in “pure” psychiatric cases: not pathological suicidality, but excessive paternalism (1, 4, 6). In the presence of irreversible somatic illness, overemphasis on a distant or well-controlled psychiatric comorbidity can lead to unjustified denial of autonomy, transforming vulnerability protection into clinical paternalism (4, 9). The literature suggests that while the evaluative threshold should remain rigorous, it must not be indiscriminately raised but calibrated according to the actual causal role of the psychiatric disorder in the EAS request (2, 3, 9). Even in this scenario, documented longitudinal assessment and regulated family involvement remain important. Psychiatric history reconstruction allows for demonstration of clinical stability and absence of significant relapses, while family input may offer insights into the consistency of the request and the evolution of personal values (3, 40, 54). However, as noted, such contributions must be carefully managed from a medico-legal standpoint to prevent undue pressure or interference in the decision-making process (4, 22, 23). Overall, the literature suggests that in cases of somatic comorbidity, the ethical and clinical approach to EAS must be guided by a different balance than in cases where mental disorder is the sole motivation (4, 9). The priority is not to raise the evaluative threshold indiscriminately, but to avoid exclusionary or discriminatory criteria based on psychiatric diagnosis (2, 4, 6). In this context, psychiatry plays an essential role in safeguarding fairness in the decision-making process, helping to avoid both excessive permissiveness and excessive protectionism (3, 4, 9).

## Conclusions

The integrated analysis of the literature confirms that EAS in psychiatric settings represent a domain where individual self-determination, clinical vulnerability, evaluative complexity, and medico-legal responsibility often intersect in a state of tension (2–4, 9). Unlike irreversible somatic illnesses, mental disorders display highly variable trajectories, frequently unpredictable treatment responses, and a subjective dimension of suffering that is difficult to quantify objectively (3, 16). For these reasons, every EAS request in psychiatry requires greater methodological caution and a more complex evaluative framework than other end-of-life contexts (1, 3, 4). The review highlights that no single clinician should bear the sole responsibility for determining decision-making capacity, irremediability of the disorder, and motivational coherence of the request (3, 7, 22). The adoption of multidisciplinary commissions emerges as the most robust and coherent solution to address the complexity of this phenomenon (3–5, 9). A structured evaluative model should include at least two psychiatrists, one of whom must be independent, a forensic physician, and, when appropriate, a specialist in any coexisting somatic condition (4, 7, 22, 23). Within this evaluative framework, the involvement of an internist or geriatrician may also be appropriate in cases where the mental disorder is the primary motivation for the request, not to redirect the assessment to somatic illness, but to ensure a comprehensive evaluation of general health status, functional autonomy, physical comorbidities, and overall impact on quality of life (36–39). This contribution prevents the assessment of mental suffering from being isolated from the broader clinical context and reinforces the principle of proportionality in decision-making, integrating the psychiatric perspective into a wider analysis of the person (4, 9). Within this framework, the forensic physician plays a central role in ensuring procedural integrity by verifying the consistency among clinical evaluations, normative criteria, and produced documentation, as well as the medico-legal sustainability of the entire evaluative process (4, 22, 23). An equally crucial principle concerns the need to avoid discrimination against patients affected by mental disorders. When an EAS request is motivated by a terminal somatic illness or another non-psychiatric condition, the mere presence of a psychiatric diagnosis cannot and must not constitute grounds for exclusion (2, 4, 9). Decision-making capacity must be assessed in a decision-specific and time-specific manner, not diagnosis-specific: what matters is not the diagnostic label, but the quality of reasoning, stability of will, and motivational coherence with respect to the decision in question (13–15, 33). Automatic exclusion would introduce a form of structural inequality unsupported by scientific evidence and contrary to principles of justice and fairness (2, 4, 6). At the same time, the review shows how fragile and elusive the concept of “irremediability” of mental suffering remains. Available evidence indicates that patients previously considered “treatment-resistant” may respond to new interventions, including innovative pharmacological treatments, intensive psychological therapies, neuromodulation, and advanced psychosocial support programs (16–19). This necessitates that EAS be considered only after a truly exhaustive, documented therapeutic pathway that respects the best available practices (3, 16). Mental suffering must not become the result of inadequate resources, social isolation, poverty, or therapeutic abandonment: responding with EAS to conditions reflecting healthcare system failures would constitute an ethical failure before a clinical one (4, 5, 9). Overall, this review suggests that a responsible regulation of EAS in psychiatry cannot merely replicate models developed for somatic conditions (3, 9). A specific system is needed, built around multidisciplinary, longitudinal, documented, and rigorous evaluation, capable of addressing the complexity of mental suffering and ensuring that the request is not the manifestation of an unrecognized or insufficiently treated need (3–5, 7). Only through this approach can it be ensured that EAS, where permitted, represents a truly autonomous, considered, and morally justifiable decision (2, 4, 9). Ultimately, the challenge is not to oppose autonomy and protection, but to create a model capable of allowing them to coexist in a balanced and responsible way. A system based on collegial assessment, non-discrimination, clinical proportionality, procedural transparency, and maximal therapeutic commitment represents the only path to addressing the inherently sensitive issue of euthanasia and medically assisted suicide in psychiatric contexts with clinical rigor, ethical responsibility, and medico-legal sensitivity (3, 4, 9).

## Data Availability

The original contributions presented in the study are included in the article/supplementary material. Further inquiries can be directed to the corresponding author.
